# Editorial Department

**Published:** 1856-11

**Authors:** 


Dr. Forbes, of London, has proposed the theory that the last image impressed on the retina is there preserved in cases of sudden death. We find, in an Auburn paper, the following account of an examination of the eye of Mr. Beadle, found murdered in the streets of that city a few weeks ago. We do not know with how much care or ability the experiment was conducted, and some of the statements relative to the effect of atropine upon the dead eye seem to us very improbable, but our readers can read and judge for themselves:

"*Singular Optical Experiment.*—From the circumstance of reading Dr. Forbes' system of examination in the case of murder, which appeared in several of our papers, a few months ago, we have been induced to exercise a similar experiment on the eye of the unfortunate Beadle, and trust the result will induce some of your readers to make the like experiment on the eyes of the brute creation.

"At first we suggested the saturation of the eye in a weak solution of atropine, which evidently produced an enlarged state of the pupil. On observing this, we touched the end of the optic nerve with the extract, when the eye instantly became protuberant. We now applied a powerful lens, and discovered in the pupil the rude worn-away figure of a man, with a light coat, beside whom was a round stone, standing or suspended in the air, with a small handle stuck as it were in the earth. The remainder was debris, evidently lost, from the destruction of the optic nerve, and its separation from the mother brain. Had we performed this operation when the eye was entire in the socket, with all its powerful connection with the brain, there is not the least doubt but that we should have detected the last idea and impression made on the mind and eye of the unfortunate man. The thing would evidently be entire; and perhaps we should have had the contour, or better still, the exact figure of the murderer. The last impression before death is always more terrible on the brain from fear than from any other cause; and figures impressed on the pupil more distinct, which we attribute to the largeness of the optic nerve, and its free communication with the brain. We believe the brain is more intimately connected with vision, than either with sense of taste, hearing or feeling; and from this very reason, that we are constantly seeing a variety of objects, giving exercise to the brain for the same quantity of idea.

"Thomas Bellamy, M. D.
"C. P. Sanford, M. D."

